# Physical activity, cognition and academic performance: an analysis of mediating and confounding relationships in primary school children

**DOI:** 10.1186/s12889-018-5863-1

**Published:** 2018-07-31

**Authors:** Adrian McPherson, Lisa Mackay, Jule Kunkel, Scott Duncan

**Affiliations:** 1 School of Sport and Recreation, Auckland University of Technology, Private Bag 92006, Auckland, 1142 New Zealand; 2 Institute for Sports and Sports Science, Karlsruhe Institute of Technology, Karlsruhe, Germany

**Keywords:** Physical activity, Cognition, Academic performance, School, Children, Mediation, SEM, Multigroup analysis

## Abstract

**Background:**

Exploring the relationship between physical activity, cognition and academic performance in children is an important but developing academic field. One of the key tasks for researchers is explaining how the three factors interact. The aim of this study was to develop and test a conceptual model that explains the associations among physical activity, cognition, academic performance, and potential mediating factors in children.

**Methods:**

Data were sourced from 601 New Zealand children aged 6–11 years. Weekday home, weekday school, and weekend physical activity was measured by multiple pedometer step readings, cognition by four measures from the CNS Vital Signs assessment, and academic performance from the New Zealand Ministry of Education electronic Assessment Tools for Teaching and Learning (e-asTTle) reading and maths scores. A Structured Equation Modelling approach was used to test two models of variable relationships. The first model analysed the physical activity-academic performance relationship, and the second model added cognition to determine the mediating effect of cognition on the physical activity-academic performance association. Multigroup analysis was used to consider confounding effects of gender, ethnicity and school socioeconomic decile status.

**Results:**

The initial model identified a significant association between physical activity and academic performance (*r* = 0.225). This direct association weakened (*r* = 0.121) when cognition was included in the model, demonstrating a partial mediating effect of cognition. While cognition was strongly associated with academic performance (*r* = 0.750), physical activity was also associated with cognition (*r* = 0.138). Subgroups showed similar patterns to the full sample, but the smaller group sizes limited the strength of the conclusions.

**Conclusions:**

This cross-sectional study demonstrates a direct association between physical activity and academic performance. Furthermore, and importantly, this study shows the relationship between physical activity and academic performance is supported by an independent relationship between physical activity and cognition. Larger sample sizes are needed to investigate confounding factors of gender, age, socioeconomic status, and ethnicity. Future longitudinal analyses could investigate whether increases in physical activity can improve both cognition and academic performance.

## Background

Over the course of the last century, a multidisciplinary field of knowledge has developed that has identified several cognitive and academic benefits of regular physical activity (PA) [1–7]. The idea that PA can enhance cognitive and academic ability has consequently received significant attention in health and education fields [8–10]. It is recognised that PA triggers change in the human brain due to increases in metabolism, oxygenation and blood flow providing hormones that promote neurological health [11, 12]. Those changes are particularly important for the developing paediatric brain [9, 12]. Researchers are now clarifying how relationships between PA and cognition interact to guide the best way forward to promote neurological, cognitive and academic benefits for children.

Sibley and Etnier completed a meta-analysis of 44 studies into the relationship between PA and cognitive abilities [6]. They found all included studies reported significant and positive effects of PA within physical education (PE) and cognition in youth, regardless of the study design and type of PA [6]. The greatest effects were seen with perceptual skills and academic readiness tests [6]. A review of 17 studies by Trudeau and Shepherd on the impact of PA on academic performance of children in primary and secondary school also found positive relationships between PA and school results [7]. Combined analysis of the seven quasi-experimental studies showed that the enriched PE programmes demanded a substantial reduction in the time allocated for academic tuition but academically children achieved at least equally despite the reduced teaching time [7]. Ten cross-sectional studies showed positive association between PA and academic performance [7]. Despite concurrence about a positive relationship between PA and cognition, both reviews note limitations due to the small number of true experimental studies and by potential confounding variables. For example, Sibley and Etnier found 57 different methods of cognitive assessment used by investigators, many with poor or unknown psychometric properties [6]. In another meta-analysis, Hillman et al., completed a review of 14 studies examining PA and neuroelectric concomitants of cognition during childhood [4]. They found PA and cardiovascular fitness have short and medium-term benefits for neurocognitive performance in youth [4]. The studies used laboratory measures to measure neurological activity on subjects performing a range of cognitive tasks and formal assessments. They found increased fitness and PA improve cognitive function and brain health, with higher-fit children demonstrating attributes such as greater attention, faster information processing, and higher scores in standardised achievement tests. Only one study which provided neutral findings did not show any improvement in cognitive function.

Furthermore, two detailed studies from 2016 provide strong support for the relationship PA has with cognition and academic performance [13, 14]. For the Copenhagen Consensus, 24 researchers from eight countries met to reach an evidence-based consensus on the effects of PA on children and youths aged 6–18 years [13]. The authors concur that PA and cardiorespiratory fitness are beneficial to brain structure, brain function and cognition in children and youth [13]. They advise that PA before, during and after school promotes scholastic performance in children and youth, with even a single session of moderate PA having an acute benefit to brain function, cognition and scholastic performance [13]. In the other study, eight key researchers in the PA-cognition field started from a database of 6237 articles and identified 137 key articles to consider [14]. The review focused on two specific questions: Among children age 5–13 years, do PA and physical fitness influence cognition, learning, brain structure, and brain function? And among children age 5–13 years, do PA, PE, and sports programs influence standardized achievement test performance and concentration/attention? They found promising results showing relations among PA, cognition, brain structure, and brain function, with no negative effects on children. The 26 cross-sectional and cohort-based studies involving PA provided positive support for the relationship between PA and cognitive function, with greater amounts or enhanced forms of PA being associated with greater improvements in cognitive function [14]. For the second question, the authors stated the studies of acute PA interventions had mixed results, likely owing to the differences in tasks administered, the nature of the task, and the type of PA [14]. However, authors advise a number of methodological weaknesses including a lack of information about estimates of random variability in the outcome data, information about the time of day at which the cognitive measures were assessed was not provided, varying and inconsistent measures of fitness and academic performance, and poor control of confounders. They particularly noted many studies did not give statistical power of the findings, including 95% of the studies relating to the second question [14].

The analyses above show that excellent research has established that PA is associated with both cognition and academic performance for children. However, few studies have investigated how the three areas of PA, cognition and academic performance interact. Does a relationship between PA and cognition necessarily lead to better academic performance? Does an independent relationship between PA and academic performance relationship exist, or does it act through cognition? Is the PA-cognition-academic performance relationship the same for different groups of children? Specifically, does the relationship between PA and academic performance remain once cognition is accounted for? Therefore, the aim of this study is to develop and test a conceptual model that explains the cross-sectional associations among PA, cognition and academic performance in children aged 7–10 years.

## Methods

### Participants

A total of 675 participants (326 male, 349 female) were part of an eight-week randomised controlled trial: *Healthy Homework* was a curriculum-based, classwork and homework schedule designed to promote PA and healthy eating [15]. This study analyses data collected from participants at baseline, prior to receiving any intervention. Eligibility criteria for the schools were as follows: a school with more than 100 students, location within Auckland or Dunedin cities, and a contributing, full primary, or composite structure that included at least one class each of students in school years 3–5. A total of 16 primary schools from Auckland (*n* = 10) and Dunedin (*n* = 6) were selected to participate in the study. Socioeconomic decile ratings of participating schools ranged from 3 to 10 (median [IQR] = 8 [6, 9]). Decile is a New Zealand Ministry of Education rating system for school funding based on SES with 1 being low and 10 being high. Decile reflects the extent to which the school draws their students from low socio-economic communities, rather than the SES mix of the school or individual students. For example, low decile schools have the highest proportion of students from low socio-economic communities. Students were selected to participate from one Year 3, one Year 4, and one Year 5 class from each school; simple random sampling was used in instances where there were two or more classes per year. Written parental consent and student assent were obtained for children to participate in the study. Ethical approval was obtained from the Auckland University of Technology Ethics Committee (10/159).

### Measures

Demographic information was obtained from the school records and included gender, age, school, ethnicity and socioeconomic decile. All demographic variables were partitioned into groups: Decile (Low 1–5, Mid 6–8, and High 9–10); School year (Year 3, Year 4, and Year 5); and Ethnicity (New Zealand European and Non-New Zealand European). Ideally, socioeconomic decile groupings would be 1–3, 4–7, and 8–10, and a greater range of specific ethnic groups would be analysed, but the total numbers for lower socioeconomic decile students and non New Zealand European ethnic groups were too small for multi-group analysis to be completed (Table 1).

**Table 1:** Descriptive statistics of 601 subjects used in analyses

Age									
	Male			Female			Total		
	N	M + SD	Min + Max	N	M + SD	Min + Max	N	M + SD	Min + Max
School Year 3	91	7.74 ± 0.548	6.78, 8.75	96	7.65 ± 0.661	6.48, 9.25	187	7.69 ± 0.609	6.48, 9.25
School Year 4	103	8.68 ± 0.598	7.60, 9.86	105	8.76 ± 0.593	7.61, 9.89	208	8.72 ± 0.595	7.60, 9.89
School Year 5	106	9.62 ± 0.521	8.11, 10.8	100	9.77 ± 0.537	8.75, 10.8	206	9.70 ± 0.534	8.11, 10.8
Total	300	8.73 ± 0.942	6.78, 10.8	301	8.74 ± 1.05	6.48, 10.8	601	8.74 ± 0.995	6.48, 10.8
Ethnicity									
	Male (*n* = 300)			Female (*n* = 301)			TOTAL (*n* = 601)		
Maori	17 (5.7%)			22 (7.3%)			39 (%6.5)		
Pacific Island	12 (4%)			13 (4.3%)			25 (4.2%)		
Asian	34 (11.3%)			65 (21.6%)			99 (16.5%)		
Other	8 (2.7%)			11 (3.7%)			19 (3.2%)		
NZ European	229 (76.3%)			190 (63.1%)			419 (69.7%)		
Total	300 (100%)			301 (100%)			601 (100%)		
Decile									
Decile 3	23 (7.7%)			18 (6.0%)			41 (6.8%)		
Decile 4	7 (2.3%)			13 (4.3%)			20 (3.3%)		
Decile 5	14 (4.7%)			33 (11.0%)			47 (7.8%)		
Decile 6	42 (14.0%)			47 (15.6%)			89 (14.8%)		
Decile 7	44 (14.7%)			44 (14.6%)			88 (14.6%)		
Decile 8	61 (20/3%)			46 (15.3%)			107 (17.8%)		
Decile 9	46 (15.3%)			38 (12.6%)			84 (14.0%)		
Decile 10	63 (21.0%)			62 (20.6%)			125 (20.8%)		
Total	300 (100%)			301 (100%)			601 (100%)		
School year									
Year 3	91 (30.3%)			96 (31.9%)			187 (31.1%)		
Year 4	103 (34.3%)			105 (34.9%)			208 (34.6%)		
Year 5	106 (35.3%)			100 (33.2%)			206 (34.3%)		
Total	300 (100%)			301 (100%)			601 (100%)		

PA was assessed using sealed NL-1000 pedometers (New Lifestyles Inc., Lee’s Summit, MO) over five consecutive days (three weekdays, two weekend days). NL-1000 pedometers have a multiday memory function that automatically stores step counts by day of week for up to seven days. Previous research has established the validity of these pedometers for measuring steps in children [16]. Two pedometers were assigned to each child: one clearly labelled ‘School’ and the other ‘Home’. The ‘School’ pedometer was worn during school hours, while the ‘Home’ pedometer was left inside a collection tray in the classroom. At the conclusion of the school day, each child placed their ‘School’ pedometer in the tray and attached their ‘Home’ pedometer. Upon arrival at school the next day, the teacher reminded the children to switch over their pedometers again. This resulted in three measures of PA: average weekday steps at home, average weekday steps at school, and average steps at weekend.

The cognitive abilities of children were measured using CNS Vital Signs (CNSVS): a standardised cognitive screen assessment suitable for participants aged 7–90 years [17]. CNSVS is a web-based assessment battery with seven tests that are scored individually and combined to give scores in nine different areas. Four of the nine CNSVS measures were considered for this study: Composite Memory (recognize, remember, and retrieve words and geometric figures), Executive Function (recognize rules, categories, and manage or navigate rapid decision making), Psychomotor Speed (perceive, attend, respond to complex visual-perceptual information and perform simple fine motor coordination), and Reaction Time (react, in milliseconds, to a simple and increasingly complex direction set) [18]. The other domains could not be used because of the difficulty in administering the Complex Attention Test, and the four remaining domains used combinations of the same base assessment.

Academic performance was measured using the NZ Ministry of Education electronic Assessment Tools for Teaching and Learning (e-asTTle). The e-asTTle assessments have more than 2000 curriculum based assessment items standardised on over 50,000 students covering curriculum levels 2—4 to assess student’s achievement and progress in reading, writing and mathematics and the Maori equivalents of panui, tuhituhi, and pangarau [19–22]. Assessments can be completed at any time during the school year. Measures are norm-referenced and used to evaluate children’s progress through the school year [20]. Regardless of items present in a test, e-asTTle can be used to compare progress and performance within students and between students to that of national norms and curriculum achievement objectives and levels [21]. Teachers create their own multi-choice assessment as the e-asTTle software generates a test that selects the best set of items meeting the teacher’s content and difficulty constraints [21]. For this study, researchers set up e-asTTle questions for reading with 10 questions and maths with 12 questions. Thus, raw scores ranged from 0 to 10 for reading, and 0–12 for maths. A research team conducted both the reading and maths assessments, which were done using pen and paper within a 10-min time limit. Researchers marked total scores, and results were entered into a computer by research assistants. The e-asTTle software converts raw scores into measures that align with a child’s curricular needs [21]. Raw scores were sufficient for the current analyses because they give a measure of academic performance for students in relation to peers of the same school year.

For each school, CNSVS and e-asTTle baseline measures were collected by a team of researchers on one day. Pedometers were issued to children and height and weight measures taken on a separate day within a month by trained researchers. CNSVS assessment was completed before the e-asTTle test, with at least 30 min between the two. The CNSVS assessment was conducted in groups using school computer facilities or libraries and assisted by at least three researchers. Group sizes and types of computers depended on the facilities and computers provided by the school. Researchers introduced the test beforehand while instructions for each test appeared on the screen before each test started. Researchers were available for the children in case they did not understand the instructions or if children clicked it away too quickly. CNSVS was introduced for the research purposes and is not part of routine school assessment practice.

The e-asTTle assessments were introduced and explained by the researchers. The assessment started with a two minute reading attitude questions, but those are not included in this analysis. The reading test was then conducted before the math test with a time limit of 10 min. Students are used to e-asTTle assessment as part of normal school assessment procedures undertaken throughout the academic year. However, this testing was not part of school assessment procedures, and schools did not receive any data from e-asTTle assessment.

### Statistical analysis

All variables were checked for normality, skewness and outliers. The distribution of the CNSVS composite memory item was skewed positively, but that reflects what is to be expected in the general population thus data were not transformed [17]. The other CNSVS measures were normally distributed. The two e-asTTle and three pedometer variables were normally distributed with no problematic outliers.

To analyse the data, this study used Structured Equation Modelling (SEM). SEM is appropriate to test and analyse this multifaceted field as it is able to consider individual and total relationships between variables and their mediating effects. Further, it has a robust multi-group analysis process to assess model fit for subgroups to ensure valid interpretation of between group differences. Analysis was completed using bias-corrected bootstrapping (200 samples) for 95% confidence intervals.

The extent of missing values was assessed on the full study cohort. To minimize loss of data, subjects with data for at least five of the nine variables included in the model were retained. That reduced the study cohort from 675 to 601. The IBM SPSS Missing Value Analysis (MVA) was performed on the 601 participants with the Expectation Maximisation (EM) method specified to generate a data set with imputed values for Structural Equation Modelling (SEM). Based on inspection of missing data patterns and EM data imputation, data are assumed to be missing at random (MAR). Preliminary descriptive statistics were obtained for the subjects and bivariate analyses completed to screen for relationships between variables. Prior to completing an SEM analysis, a confirmatory factor analysis (CFA) was completed on the individual latent variables: PA, cognition and academic performance. The cognition latent variable demonstrated its four measured indicators provided a good fit with the data (× 2 (2) = 3.31, *p* = 0.191, RMSEA = 0.033, TLI = 0.988). The CFA model for PA and academic performance was unidentifiable due to correlated error terms in the three pedometer step indicators and two e-asTTle indicators respectively.

**Fig. 1:**
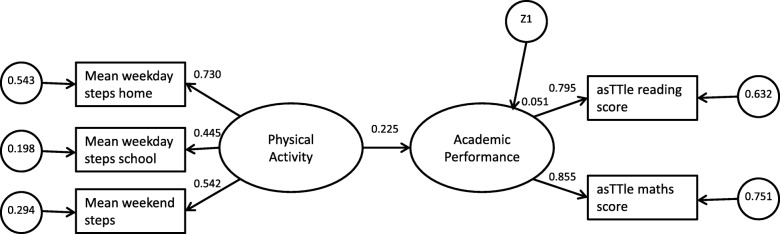
Structured Equation Model explaining the relationship between physical activity, and academic performance (Model 1), χ^2^ (4) = 13.5, *p* = .009, RMSEA = 0.063, CFI = 0.983, TLI = 0.958, PNFI = 0.391. Two variable model explaining the relationship between physical activity and academic performance

**Fig. 2:**
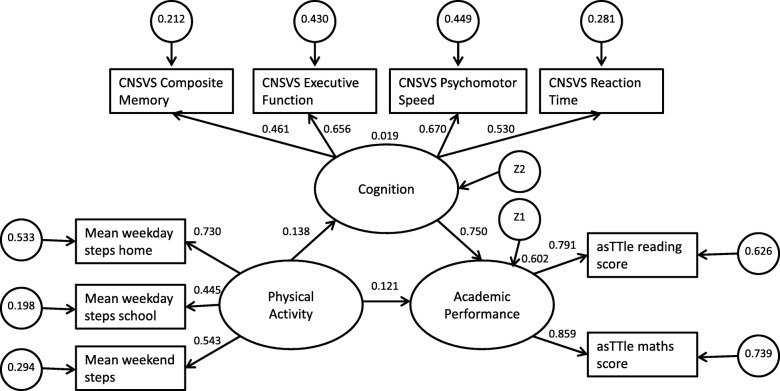
Structured Equation Model explaining the relationship between physical activity, cognition and academic achievement (Model 2), χ^2^ (24) = 67.6, *p* = .000, RMSEA = 0.055, CFI = 0.963, TLI = 0.944, PNFI = 0.628. Three variable model explaining the relationships between physical activity, cognition and academic performance

The hypothesized models are in Figs. 1 and 2. Circles represent latent variables, and rectangles represent measured variables. The hypothesized models examined the strength of association between PA, cognition and academic performance. Academic performance was considered a latent variable with two indicators: (asTTle maths and reading scores). It was hypothesized that PA (a latent variable with three indicators: mean weekday steps home, mean weekday steps school, and mean weekend steps) was associated with higher levels of academic performance (Model 1, Fig. 1). Additionally, it was hypothesized that cognition would mediate the strength of association between PA and academic performance (Model 2, Fig. 2). Cognition was a latent variable with four indicators (CNSVS Composite Memory, Executive Function, Psychomotor Speed, and Reaction Time).

## Results

### Assumptions

Data for 601 students (49.8% male) aged 6.5–10.8 years residing in New Zealand were available for analyses (Table 1). Overall, bivariate analyses demonstrate consistent small to medium significant relationships between the areas of cognition and academic performance of participants, and pedometer steps showed trivial relationships with cognition and academic performance variables.

### Structured equation modelling

Although the chi-square fit statistic for Model 1 was significant, other indicators of model fit supported good fit with the data (χ^2^ (4) = 13.5, *p* = 0.009, RMSEA = 0.063, CFI = 0.983, TLI = 0.958, PNFI = 0.391). Greater academic performance was marginally associated with higher levels of PA (standardised coefficient = 0.225, *p* < 0.001) (Table 2.). The model accounted for 5.1% variance in academic performance. The final Model 1 with standardized coefficients is in Fig. 1.

**Table 2:** Significance outputs and 95% Bias-corrected confidence intervals from SEM models

	Sample Size	Model 1				Model 2											
		PA-AP: Direct				PA-AP: Direct				PA-Cognition: Indirect				Cognition-AP			
		β	LCL	UCL	*P*	β	LCL	UCL	*P*	β	LCL	UCL	*P*	Β	LCL	UCL	*P*
Full sample	601	.225	.129	.310	< .01	.121	.008	.197	< .05	.138	.010	.274	< .05	.750	.659	.828	< .05
Male	300	.191	.044	.355	< .05	.087	−.037	.231	.181	.163	−.094	.352	.181	.716	.600	.856	< .01
Female	301	.277	.120	.460	< .01	.169	.055	.338	< .05	.147	−.046	.317	.096	.778	.664	.906	< .01
NZEuro	419	.214	.100	.352	< .01	.083	−.039	.187	.243	.177	.022	.321	< .05	.730	.620	.835	< .05
NonNZEuro	182	.189	.000	.335	.071	.179	−.002	.340	.052	.049	−.233	.272	.752	.818	.649	.939	< .05
Decile 1–5	108	.351	.086	.554	< .05	−.018	−.970	.287	.909	.398	−.076	.718	.069	.869	.521	1.496	.094
Decile 6–8	284	.146	−.094	.278	.222	.152	−.019	.278	.071	−.011	−.193	.167	.977	.706	.552	.826	< .05
Decile 9–10	209;	.198	.060	.338	< .05	.009	−.156	.182	.730	.253	.032	.448	< .05	.813	.000	.920	.056

*PA = Physical Activity; AP = Academic Performance; NZEuro = New Zealand European ethnicity; NonNZEuro = Non New Zealand European ethnicity;*

β = standardised coefficient; LCL = lower 95% confidence limit, UCL = upper 95% confidence limit using bias-corrected bootstrapping

Model 2 tests the hypothesis that some of the PA-academic performance relationship is mediated by cognition (Fig. 2). Similarly, the chi-square statistic for Model 2 was significant but other model fit indicators support good fit with the data (χ^2^ (24) = 67.582, *p* = .000, RMSEA = .055, CFI = .963, TLI = .944, PNFI = .628). The indirect association of PA on academic performance is shown in the PA–cognition pathway (standardised coefficient = 0 .138, *p* < 0.05). The total relationship between PA and academic performance is gained by adding the relationships PA-academic performance and PA-cognition (0.259). Over half (60.2%) of the variance in academic performance was accounted for by PA and cognition.

### Structured equation modelling – Subgroup analyses

For gender, measurement invariance testing revealed a lack of equivalence for the scalar and residual models for Model 1. However, Model 2 achieved appropriate fit. Model 2 shows that higher levels of PA was not significantly associated with greater academic performance in boys (standardised coefficient = 0.087, *p* = 0.222). Model 2 shows a significant small direct relationship between PA and academic performance for girls (standardised coefficient = 0.169, *p* < 0.05). For age grouping by school year, no analysis was able to be completed. Both Model 1 and Model 2 had acceptable fit indices for the configural and metric models, but the scalar and residual model fit indices were significantly lower. As such, the Models are not equivalent for different age groups and group comparison cannot be made [23].

Model fit indices for the two ethnic groups for both models were adequate across all four tests of measurement invariance. Thus, model fit is supported for comparison between ethnic groups. For New Zealand European students, Model 1 showed greater academic performance was marginally associated with higher levels of PA (standardised coefficient = 0.214, *p* < .01). Similar relationships, although not significant, were shown for non-New Zealand European students (standardised coefficient = 0.189, *p* = 0.225). Model 2 shows for New Zealand European students, PA was not associated with academic performance (standardised coefficient = 0.083, *p* = 0.171). However, PA was associated with cognition (standardised coefficient = 0.177, *p* < 0.05). None of the PA pathways in Model 2 were significant for non-New Zealand European students (Table 2).

For socioeconomic decile groupings, model fit indices for both models were adequate across all four tests of measurement invariance. For students from low socioeconomic decile schools, Model 1 showed a moderate significant relationship between PA and academic performance (standardised coefficient = 0.351, *p* < 0.05 and standardised coefficient = 0.198, *p* < 0.05, respectively). The relationship was not significant for students in the mid socioeconomic decile schools (standardised coefficient = 0.146, *p* = 0.167). For students from low socioeconomic decile schools, Model 2 showed no significant relationship between PA and academic performance (standardised coefficient = 0.018, *p* = 0.909), or between PA and cognition (standardised coefficient = 0.398, *p* = 0.141). For students from mid socioeconomic decile schools, Model 2 shows a small significant relationship between PA and academic performance (standardised coefficient = 0.152, *p* < 0.05), but not between PA and cognition (standardised coefficient = − 0.011, *p* = 0.904). Lastly, students from high socioeconomic decile schools showed no significant relationship between PA and academic performance in Model 2 (standardised coefficient = 0.009, *p* = 0.887); however, there was a small significant relationship between PA and cognition (standardised coefficient = 0.253, *p* < 0.01).

## Discussion

One of the key outstanding questions in the PA-cognition-academic performance relationship is whether the association between PA and academic performance relationship is independent or if it is mediated by cognitive ability. This study explains three aspects to the relationships: (1) the individual relationships PA has with cognition and academic performance; (2) the mediating effect of cognition on the PA-academic performance relationship; and (3) the overall relationship between PA, cognition and academic performance.

This cross-sectional study supports the growing body of research showing consistent positive relationships between PA and cognition [2, 4, 14], and between PA and academic performance [7, 24, 25]. A key focus of this study was to examine those relationships further, to determine whether the association between PA and academic performance remained after considering cognition. As hypothesized for the full sample, cognition was shown to reduce the strength of association between PA and academic performance. Further, the mediating effect of cognition on the association between PA and academic performance is only partial as a small significant relationship between PA and academic performance remained. Additionally, when considering the positive association between PA and cognition, the total association between PA and academic performance is greater in Model 2 than Model 1.

The present findings differ from a similar model tested by van der Niet et al., that characterised the relationship between physical fitness, executive function and academic achievement in 263 children (145 boys, 118 girls) aged 7–12 years in the Netherlands [26]. In their two-variable model of physical fitness and academic achievement, the relationship was slightly greater than the equivalent PA-academic performance relationship for Model 1 in this study. When adding executive function to their model, they also found a stronger relationship in the physical fitness-executive function than PA-cognition for Model 2 in this study. However, the main difference was in considering mediating effects. By adding executive function, the physical fitness-academic achievement relationship dropped completely, showing a complete mediating effect of executive function [26]. Model 2 in this study, however, only found a partial mediating effect of cognition. In other words, van der Niet et al., found that executive function explained all of the variance in academic achievement, whereas the present study identified PA and cognition had independent relationships with academic performance. The differences between the two studies may be due to a number of factors including indicator measures used in assessment and participant differences. The key for both studies is they further help explain essential considerations in the PA cognition field by demonstrating the different level executive function and cognition affect the PA-academic performance relationship and the importance of considering independent and mediating effects of variables.

The present study also considered whether the hypothesized models were equivalent for demographic sub-groups (gender, age, ethnicity and school socioeconomic decile). However, due to small sub-group sample sizes, a lack of statistically significant results across the multigroup analyses precludes robust conclusions across all groups. Research in larger samples may improve the power to detect differences between groups. Previous research has shown that the relationships between PA, cognition and academic performance may differ by gender. For example, in a retrospective analysis of 5316 children, Carlson et al., found that girls in a high activity group performed better academically than those in medium and low activity groups [27]. No differences between groups were noted for boys [27]. Other studies have identified PA-cognition and PA-academic performance relationships for children of many different ethnic origins including US [27], Dutch [26], French [10], Australian [28], and Taiwanese [29], but none were identified that consider differences in the relationship on the basis of ethnicity. SES or school socioeconomic decile is the last confounding variable this study considered. Although SES is recognised as one of the main influences on children’s academic success [7, 24, 30], none of the papers reviewed for this study were shown to adjust or consider for SES. Also, this study’s use of a school-level socioeconomic decile as a measure of SES may not fully elucidate the effect of this confounding indicator. Future studies should incorporate the two important confounders of an individual-level SES indicator and the educational level of parents.

The measures used in this study have potential limitations. Pedometers give a valid and reliable indicator of overall volume of physical activity and have been used widely among student populations [16], but do not consider the intensity of the steps or time of day. High intensity aerobic activity and activity immediately prior cognitive assessment have been linked to greater cognitive function and academic performance [2, 4, 8]. Furthermore, pedometers do not monitor other aspects of fitness that have been linked to cognitive function such as acute effects of activity, cardiorespiratory fitness, resistance exercise, or combinations of exercise and activity [8, 24, 29]. Similarly, the two e-asTTle measures are well researched and robust, but additional school-based assessments such as writing could provide greater insights to children’s academic performance. Although CNSVS showed consistent strong relationships between its different measures and with academic measures both at bivariate and SEM analysis stages, the CNSVS measures used in this study do not consider all areas of cognition. Students were not familiar with the CNSVS assessment and thus results may reflect this unfamiliarity rather than difficulty with the cognitive demands and content. Differences in PA, cognition and academic performance due to age is an important potentially confounding factor for analysis [8, 12]. Accordingly, this study aimed to analyse children by school year. However, the school year multigroup analysis was not able to proceed due to a lack of measurement equivalence. Also, classroom behaviour is also shown to have a strong influence on a child’s cognition and academic performance [7, 10, 25, 28]. Initially behaviour was considered for the model, however the behaviour indicator variables had a high degree of missing values and were thus removed from the model. A final limitation is that while this study concludes a positive association between PA and cognition, and PA and academic performance, it cannot ascribe direction in those relationships. Although the theoretical assumption is that increased PA leads to better cognition and academic performance as indicated in the models, bidirectional relationships are possible, and high levels of cognition and academic performance may lead to increased PA. One of the key questions in the PA/cognition field is: are smart children active or does being active make children smart? Future studies need to ensure enough participants to enable subgroup analysis to consider confounding factors. Furthermore, longitudinal research is needed to examine PA, cognitive and academic changes over time which will provide clearer understanding to possible causal relationships.

## Conclusions

This study shows a positive association between PA and academic performance for the whole study cohort. Importantly, this study further shows that relationship remains when considering the mediating effect of cognition. Thus, the model tested identifies PA is associated with academic performance directly and indirectly through cognition. Studies with larger sample sizes are needed to investigate important confounding factors such as gender, age, SES and ethnicity.

## Abbreviations

CFA: Confirmatory Factor Analysis
CFI: Comparative Fit Index
CNSVS: CNS Vital Signs
EM: Estimation maximization
MAR: Missing at random
MCAR: Missing completely at random
MNAR: Missing not at random
MVA: Missing values analysis
NonNZEuro: Non-New Zealand European ethnicity
NZEuro: New Zealand European ethnicity
PA: Physical Activity
PE: Physical education
PNFI: Parsimonious Normed Fit Index
RMSEA: Root mean square error of approximation
SEM: Structured Equation Modelling
SES: Socio-economic status
TLI: Tucker-Lewis Index
